# Data mining cluster analysis on the influence of health factors in Casemix data

**DOI:** 10.1186/1472-6963-12-S1-O3

**Published:** 2012-11-21

**Authors:** Harleen Kaur, Ritu Chauhan, Syed Mohamed Aljunid

**Affiliations:** 1 United Nations University International Institute of Global Health (UNU-IIGH), Malaysia; 2 Hamdard University, New Delhi, India

## Background

This study explores potential data mining applications in the Casemix context, which is expected to yield effective and efficient health care services. The objective of work focuses on determining hidden relevant patterns which can’t be processed by human capabilities all alone. California Drug and Alcohol treatment Assessment (CALDATA) of administrative type database can be relevant study for the medical diagnosis in usage of alcohol and drugs for patients admitted and discharged during the stay in hospital to discover knowledge for recovery process.

## Methods

We utilized the observational study on cases registered to California Department of Alcohol and Drug Programs (ADP) to promote the initiative for increasing availability of abusive drug usage data for better drug recovery services among the California. The cases were diagnosed with Minitab diagnostic tool to access the Casemix databases for retrieval of hidden information using data mining tools. The K means clustering having used with dendrogram to determine the possibility of existence of patient admitted and discharged on the accountability for usage of abusive substance between the years 1991-1993. The classification of data is done among the educated and uneducated class for categorized race with correlation age at the time of admission to hospital. The analysis has been performed on the patients admitted due to abusive substance usage and treatment provided during the stay in hospital and discharge status for final medical diagnosis provided to patient those have suffered for long stay during hospitalization.

## Results

There has been a tremendous increase in the incidence rate of admission cases in age group 45-49 years. The probability of over 40% cases acquiring maximum number of abusive substance exists in patients who have obtained post graduate education. The decline approximately 2.3% of criminal activities after proper diagnosis to patients with high level of alcohol dependency among the cases observed.

The total number of cases evaluated to study was 1,826 in 1991-1993; total number of features selected was 1,205 for each case diagnosed. The cases were diagnosed on the basis of admission and discharge among the prevalence of abusive substance usage. The subject was classified for different subjects such as education, age, duration of stay in the hospital, estimated reduction on criminal cases, decrement of hospital cases while the treatment provided during the stay.

We calculated the overall usage of abusive substance among the categorized race at time of admission with reference to the age. The results shows white were among the categorized age group of 17 and under has the maximum usage of abusive substance whereas native Americans are the one those who have minimum consumption of abusive substance usage. In diagnosis of longest time of stay during the treatment in hospital from day of admission to day of discharge due to abusive substance usage we have calculated the overall maximum number of prevalent cases during the year 1991- 1993, we have found that longest stay was observed for male/female aged below 17 years year and were correlated to marital status that is unmarried. The clusters in the dendrogram has been observed, where the largest cluster represents the maximum number of unmarried male/ female patients diagnosed for highest abusive substance usage, whereas the second cluster represents the second highest cases for divorced/ separated has the maximum usage of abusive substance, third cluster represent the single between the age group 21-24 years accounted for 48% cases for length of stay during hospitalization, fourth cluster represents the married cases those who were among age group 35-39 years, the minimum number of cases were studied for widowed.

## Conclusions

The integrated approach, K-means and Hierarchical Clustering technique using Minitab are well suited techniques to provide insight of health service databases. The probability of patients acquiring the abusive substance depends on several factors such as education, age, marital status and several other factors related to patients. The discharge status of these highly correlates to criminal activity and discharge status of hospitalization cases which have reduced down tremendously after providing treatment to admitted patients.

